# Structural and Electrochemical Kinetic Properties of 0.5Li_2_MnO_3_∙0.5LiCoO_2_ Cathode Materials with Different Li_2_MnO_3_ Domain Sizes

**DOI:** 10.1038/s41598-018-36593-9

**Published:** 2019-01-23

**Authors:** Songyoot Kaewmala, Wanwisa Limphirat, Visittapong Yordsri, Hyunwoo Kim, Shoaib Muhammad, Won-Sub Yoon, Sutham Srilomsak, Pimpa Limthongkul, Nonglak Meethong

**Affiliations:** 1 Materials Science and Nanotechnology Program, Department of Physics, Faculty of Science, Khon Kaen University, Khon Kaen, 40002 Thailand; 2 Synchrotron Light Research Institute, Nakhon Ratchasima, 30000 Thailand; 3 National Metal and Materials Technology Center, National Science and Technology Development Agency, Pathumthani, 12120 Thailand; 4 Department of Energy Science, Sungkyunkwan University, Suwon, 16419 Republic of Korea; 5 Institute of Nanomaterials Research and Innovation for Energy (IN-RIE), Research Network of NANOTEC- KKU (RNN), Khon Kaen University, Khon Kaen, 40002 Thailand

## Abstract

Lithium rich layered oxide xLi_2_MnO_3_∙(1−x)LiMO_2_ (M = Mn, Co, Ni, etc.) materials are promising cathode materials for next generation lithium ion batteries. However, the understanding of their electrochemical kinetic behaviors is limited. In this work, the phase separation behaviors and electrochemical kinetics of 0.5Li_2_MnO_3_∙0.5LiCoO_2_ materials with various Li_2_MnO_3_ domain sizes were studied. Despite having similar morphological, crystal and local atomic structures, materials with various Li_2_MnO_3_ domain sizes exhibited different phase separation behavior resulting in disparate lithium ion transport kinetics. For the first few cycles, the 0.5Li_2_MnO_3_∙0.5LiCoO_2_ material with a small Li_2_MnO_3_ domain size had higher lithium ion diffusion coefficients due to shorter diffusion path lengths. However, after extended cycles, the 0.5Li_2_MnO_3_∙0.5LiCoO_2_ material with larger Li_2_MnO_3_ domain size showed higher lithium ion diffusion coefficients, since the larger Li_2_MnO_3_ domain size could retard structural transitions. This leads to fewer structural rearrangements, reduced structural disorders and defects, which allows better lithium ion mobility in the material.

## Introduction

Much research has been done to develop alternative cathode materials for lithium ion batteries. Commercial LiCoO_2_ materials have a limited practical capacity of around 140 mAh.g^−1^, poor thermal stability, are expensive and relatively toxic. Moreover, LiCoO_2_ is structurally unstable, resulting in a large capacity decay as the cycle numbers increase^1–3^. Lithium rich layered oxide, xLi_2_MnO_3_∙(1−x)LiMO_2_ (M = Mn, Co, Ni, among others) cathode materials have attracted much attention as potential next generation cathode materials for lithium ion batteries. This is due to their high specific capacity, ≥250 mAh.g^−1^, with an operating voltage range of 2.0–4.8 V, leading to a high energy density that is ≥900 Wh.kg^−1^ ^2,4–6^. This cathode material family consists of two compounds, Li_2_MnO_3_ and LiMO_2_. It is generally accepted that Li_2_MnO_3_ acts as a stabilizer enabling LiMO_2_ to maintain its overall structural stability when used as a cathode material^2,4,6,7^. The large capacity in the first charge process was ≥4.4 V due to activation of Li_2_MnO_3_. The charge compensation mechanisms occurring in this Li extraction process in the high voltage region may involve irreversible oxygen release from the lattice^4,8,9^, reversible oxygen redox (O^−2^/O_2_^n−^, n = 1, 2 or 3)^10–12^, or a combination of these two mechanisms^13,14^. Thackeray *et al*.^4^ reported that this material class is a composite material. These cathode materials often present domains of Li_2_MnO_3_ and LiMO_2_ components with a high degree of structural integration in the nanoscale regime. This can be observed using high-resolution transmission electron microscopy (HRTEM). These cathode materials often reveal a phase transition from a layered Li_2_MnO_3_ component to a spinel-like structure during cycling^2,4,6,15–17^. This phase transition is believed to be a cause of the large capacity decay and dramatic voltage drop leading to a high energy density loss as cycle numbers increase. Moreover, the low electrical conductivity of the Li_2_MnO_3_ component leads to poor rate capability^18^. This indicates that the electrochemical performance of lithium rich layered oxide cathode materials is largely determined by the Li_2_MnO_3_ component. Furthermore, structural properties of these cathode materials, including cation ordering and phase separation, are significantly dependent on the synthesis methods used^19–22^.

Generally, electrochemical performance of lithium ion batteries is directly related to lithium ion transport between their cathode and anode materials. Lithium ion transport affects reversible capacity, cycling stability, and rate capability of the electrode materials. The electrochemical kinetics of lithium rich layered oxide cathode materials have been studied by a few research groups using an electrochemical impedance spectroscopy (EIS) and a galvanostatic intermittent titration technique (GITT)^23–27^. The GITT measurement was performed to examine the lithium ion diffusion coefficients of lithium rich layered oxide composite cathode materials, including 0.5Li_2_MnO_3_·0.5LiNi_0.5_Mn_0.5_O_2_^23^ and 0.5Li_2_MnO_3_·0.5LiMn_0.42_Ni_0.42_Co_0.16_O_2_^24^. The results revealed that the calculated lithium ion diffusion coefficients of these cathode materials were very small, ranging from 10^−14^ cm^2^.s^−1^ to 10^−18^ cm^2^.s^−1^ and were primarily dependent upon Li_2_MnO_3_ activation^24,27,28^. The values are much lower than those of layered cathode materials such as LiCoO_2_ (10^−7^∼10^−11^ cm^2^.s^−1^)^29^ and LiMn_1/3_Ni_1/3_Co_1/3_O_2_ (10^−9^∼10^−10^ cm^2^.s^−1^)^30^.

The 0.5Li_2_MnO_3_∙0.5LiCoO_2_ material is one type of lithium rich layered oxide cathode that has been widely studied and is considered a potential candidate for next generation high energy density cathode materials for lithium ion batteries^31–35^. However, lithium ion transport behavior of this cathode material has not been investigated. Therefore, it is essential to study the parameters that affect the electrochemical kinetics of 0.5Li_2_MnO_3_∙0.5LiCoO_2_. So, herein, we report these electrochemical kinetics in terms of lithium ion diffusion in 0.5Li_2_MnO_3_∙0.5LiCoO_2_ with various Li_2_MnO_3_ domain sizes during cycling. The galvanostatic intermittent titration technique (GITT) was used for this purpose. The Li_2_MnO_3_ domain size has a large impact on the degree of Li_2_MnO_3_ activation as well as the Li-ion transport behavior of composite-based cathode materials. This work provides a better understanding of the important parameters that influence electrochemical kinetic behaviors of lithium rich layered oxide materials for next generation cathodes for lithium ion batteries.

## Results and Discussion

### Morphology characterization

**Figure 1:**
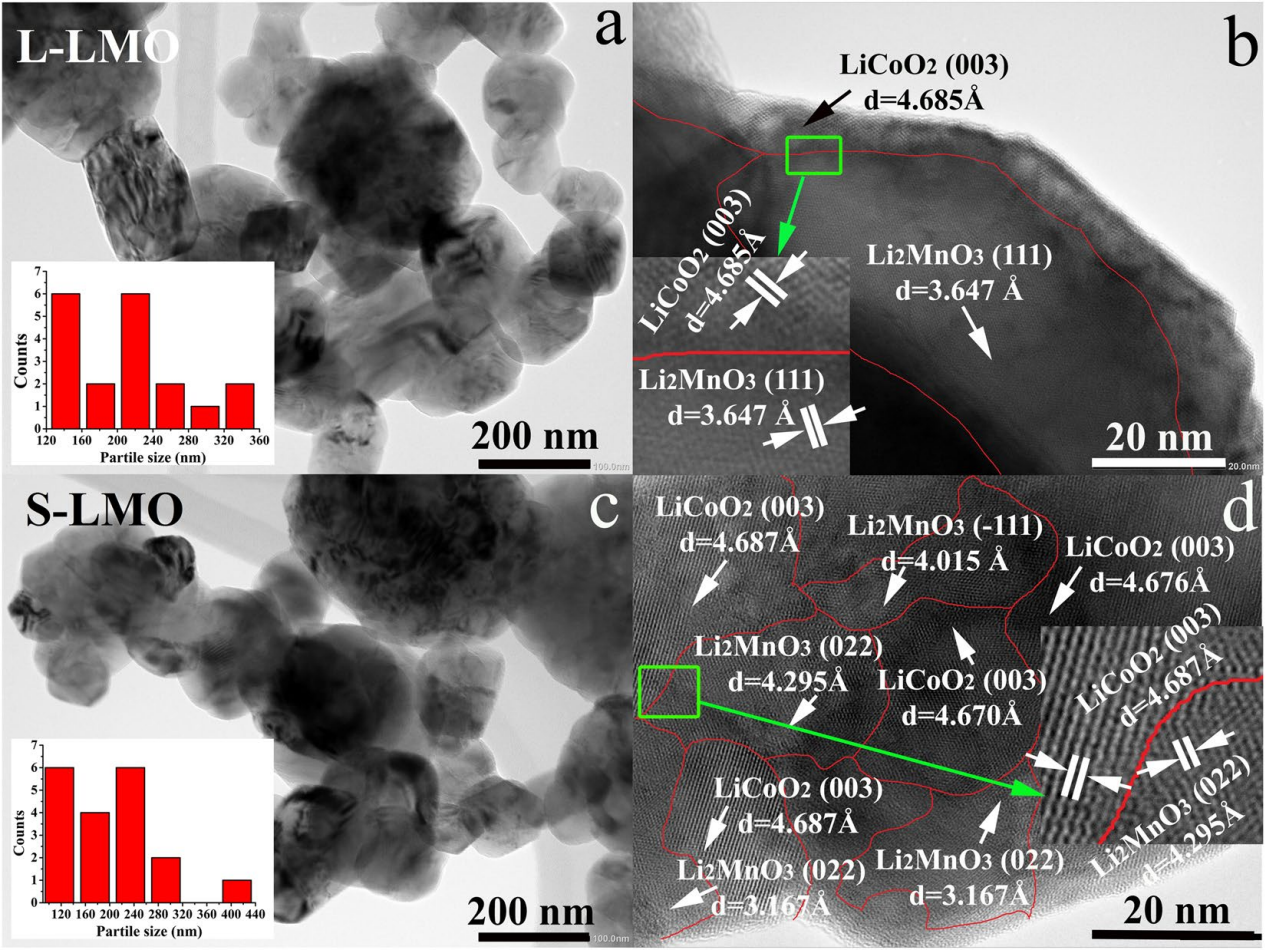
TEM and HRTEM images of the 0.5Li_2_MnO_3_∙0.5LiCoO_2_ materials with larger Li_2_MnO_3_ domain sizes (L-LMO, **a**,**b**) and the 0.5Li_2_MnO_3_∙0.5LiCoO_2_ material with smaller Li_2_MnO_3_ domain sizes (S-LMO, **c**,**d**).

High resolution transmission electron microscopy (HRTEM) was used to verify that the 0.5Li_2_MnO_3_∙0.5LiCoO_2_ materials in this study had significantly different Li_2_MnO_3_ domain sizes. Transmission electron microscopy (TEM) was used to show that both samples have similar average particle sizes of approximately 230 nm with similar particle size distributions. Their particle size distributions were quite broad, ranging from 100 nm up to 400 nm, as demonstrated in Fig. 1(a,c). The Li_2_MnO_3_ domain with a space group *C2/m* and the LiCoO_2_ domain with a space group $R\bar{{\rm{3}}}m$ were clearly observed in individual particles of both materials as demonstrated in Fig. 1(b,d). The presence of Li_2_MnO_3_ and LiCoO_2_ domains can confirm that both materials formed a composite system, consistent with previous reports^36–38^. Since the Li_2_MnO_3_ domains appear to be irregular in shape, an image processing technique employing ImageJ software, was used to determine the 2D area of individual domains. The detailed processing procedures are briefly presented as follows. We first selected images showing clear domains. We then selected a domain of interest and carefully traced it. Then, the area inside this trace was determined using the measuring function of the ImageJ software. The process was repeated 5 times/domain of interested. The 2D areas obtained from several 200 nm particle domains were then averaged. Standard deviations of the 2D area representing LMO domain were determined. The standard deviations were found to be 18% and 12% for L-LMO and S-LCO samples, respectively. Figure 1(b) shows that the L-LMO material consists of larger average Li_2_MnO_3_ domain sizes of around 2309 ± 413 nm^2^, while Fig. 1(d) reveals that the S-LMO material consists of smaller average Li_2_MnO_3_ domain sizes of about 266 ± 31 nm^2^.

The different Li_2_MnO_3_ domain sizes of the 0.5Li_2_MnO_3_·0.5LiCoO_2_ composite cathodes may be an essential parameter that significantly affects lithium ion diffusion behaviors of these cathode materials. A good understanding of the relationship between these structural characteristics and electrochemical kinetics is key to developing this cathode material class for practical applications. The impact of the Li_2_MnO_3_ domain size on the electrochemical kinetics of the 0.5Li_2_MnO_3_·0.5LiCoO_2_ material class is the central focus of this study.

### Crystal structure characterization

**Figure 2:**
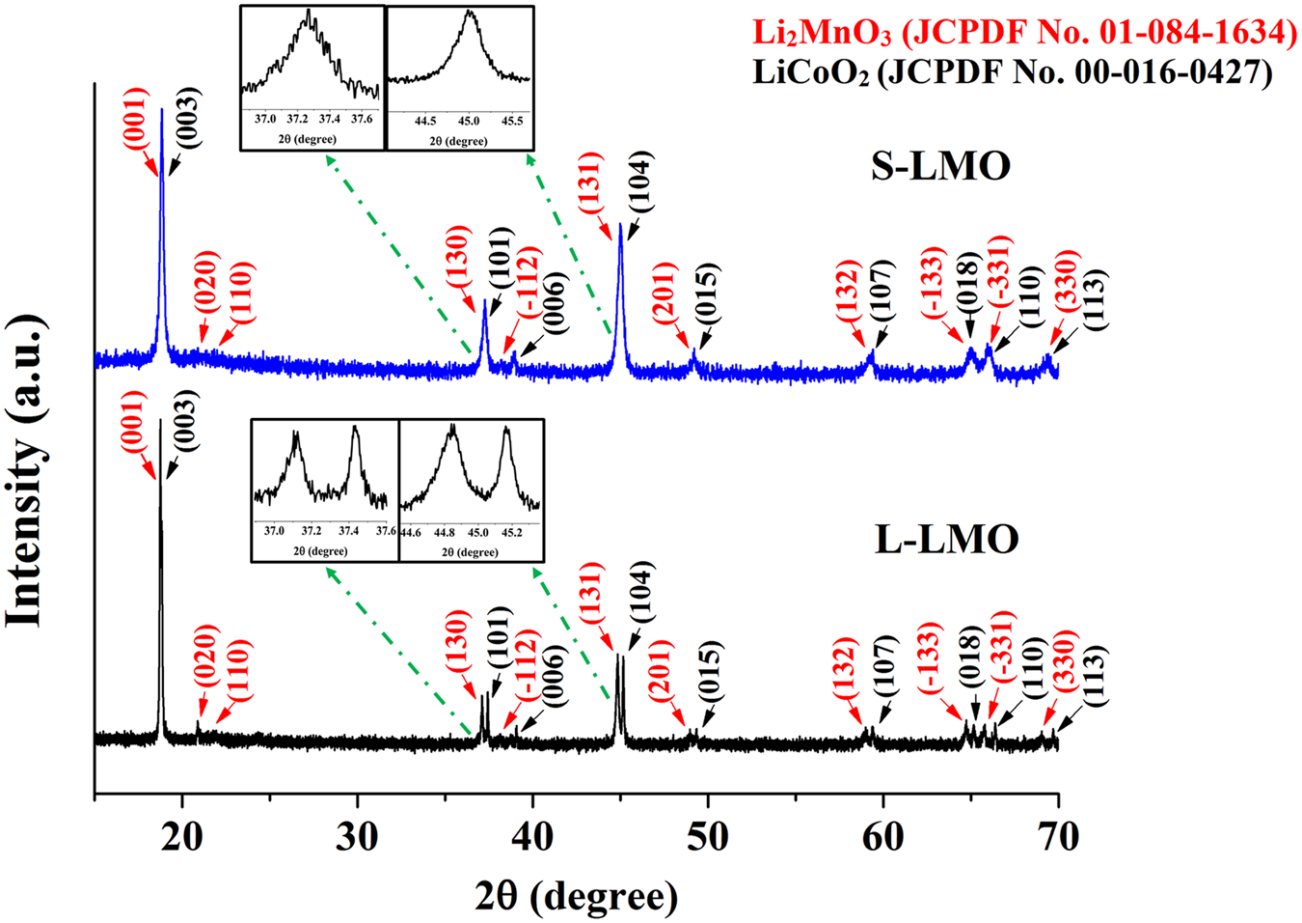
X-ray diffraction profiles of the 0.5Li_2_MnO_3_∙0.5LiCoO_2_ materials with a larger Li_2_MnO_3_ domain (L-LMO, bottom) and a smaller Li_2_MnO_3_ domain size (S-LMO, top).

X-ray diffraction (XRD) was performed to analyze the crystal structure of the S-LMO and L-LMO materials and is illustrated in Fig. 2. The diffraction peaks obtained from the materials could be indexed to both Li_2_MnO_3_ (monoclinic, space group *C2/m*) and LiCoO_2_ (rhombohedral, space group $R\bar{{\rm{3}}}m$) phases, possessing a typically layered α-NaFeO_2_ structure. Weak diffraction peaks in the 2θ range of 20°-25° correspond to the Li and Mn ion ordering in the transition metal layers of the Li_2_MnO_3_ component^39–43^ could be observed in both materials. Rietveld refinements were performed using two structural models (those of Li_2_MnO_3_ and LiCoO_2_) as illustrated in Fig. S1. The results showed that these materials had quite similar lattice constants, as demonstrated in Table S1. This suggests that the crystal structures of the Li_2_MnO_3_ and LiCoO_2_ phases in the two materials are very similar.

**Figure 3:**
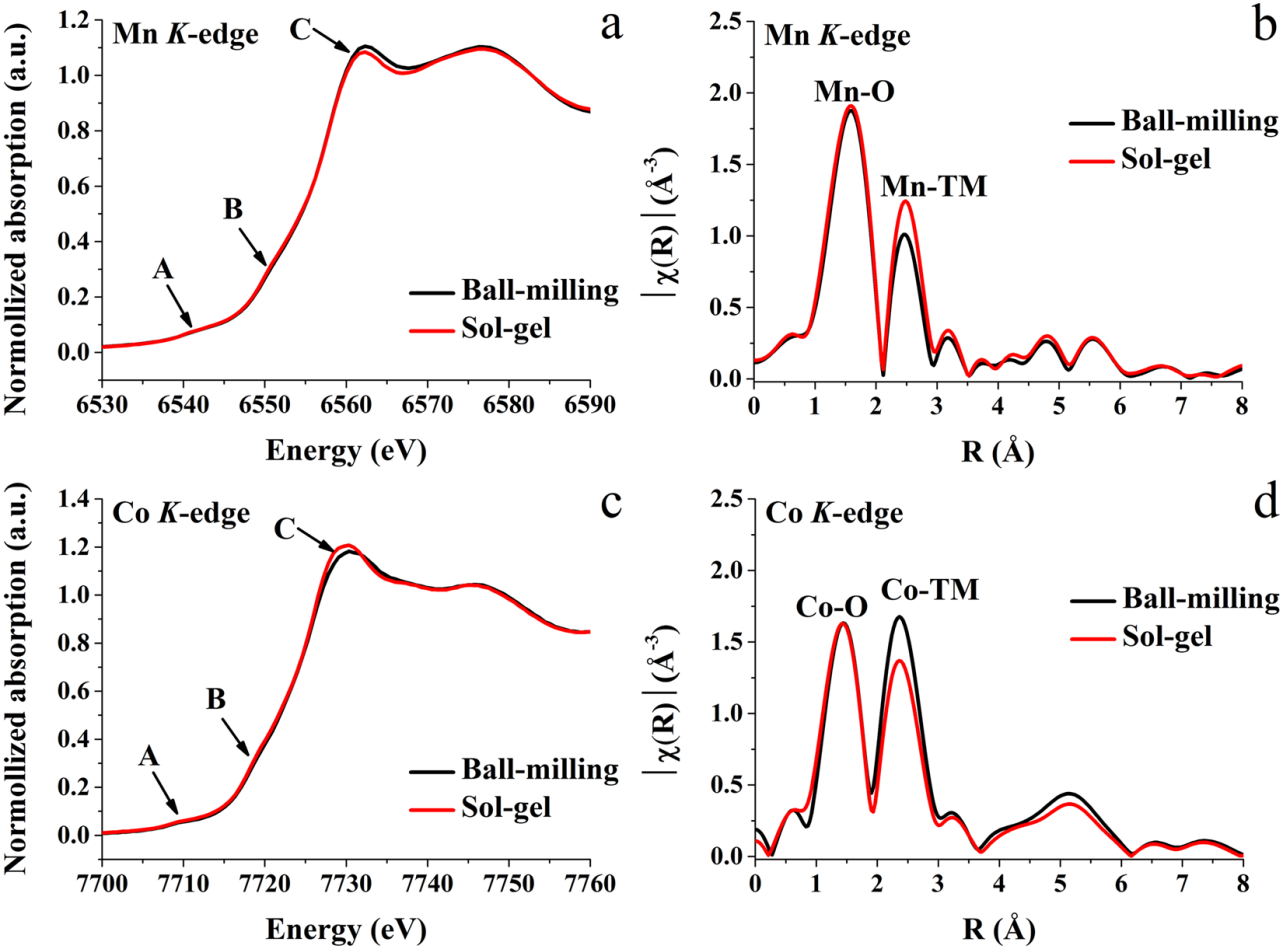
XANES spectra and *k*^2^-weighted Fourier-transformed EXAFS signals at the Mn (**a**,**b**) and Co (**c**,**d**) *K*-edges of the 0.5Li_2_MnO_3_∙0.5LiCoO_2_ materials.

The XRD peaks of the S-LMO material presented well merged XRD patterns of the Li_2_MnO_3_ and LiCoO_2_ phases. This indicates a good degree of mixing between these components as well as a peak broadening effect resulting from the nano-sized crystalline domains. The XRD peaks of the L-LMO material revealed clear separation of the XRD peaks at 2θ values above 35°. This result indicates a larger degree of separation of the Li_2_MnO_3_ and LiCoO_2_ phases^44^ as well as crystals with larger domain sizes crystals than the S-LMO material. Generally, lithium rich layered oxide materials often form a composite structure in terms of a phase separation between their Li_2_MnO_3_ and LiCoO_2_ components^4,45,46^. This result indicates that the crystal structures of the Li_2_MnO_3_ and LiCoO_2_ phases in the two materials are very similar and that their phase separation behaviors are significantly different.

### Local atomic structure characterization

X-ray absorption spectroscopy (XAS) was used to examine the local atomic structure of the 0.5Li_2_MnO_3_∙0.5LiCoO_2_ materials with various Li_2_MnO_3_ domain sizes. The XANES spectra at Mn and Co *K*-edges are presented in Fig. 3(a,c), respectively. There are three main features in the absorption spectra, including the pre-edge (feature A) and the small shoulder (feature B), and the absorption edge (feature C). The pre-edge (feature A) occurs due to the transition of electrons from a 1*s* state to an unoccupied 3*d* state. The weak shoulder (feature B) results from the transition of electrons from a 1*s* state to an unoccupied 4*p* state with a shakedown process, followed by a ligand to metal charge transfer. The main absorption edge (feature C) corresponds to the transition of electrons from a 1*s* state to an unoccupied 4*p* state without the shakedown process. The position of the main absorption edge directly relates to the oxidation states of the Mn and Co species. For Mn and Co *K*-edges, both materials presented main absorption edges in the same position, which can be confirmed from the first derivatives of their XAS spectra, as illustrated in Fig. S2, suggesting that the Mn and Co atoms in both materials possessed the same valance states. This indicated that the local environments of the Mn and Co atoms in both materials exhibited very small disordered octahedral sites^47,48^. Moreover, the XANES profiles correspond to those in previous works that used XAS to investigate the same cathode material type. They revealed that Mn and Co atoms had average valance states of 4+ and 3+, respectively^22,45,49^. As presented in Fig. 3(a,c), the normalized absorption spectra at the Mn and Co edges of both materials overlapped with few differences between them. The results suggest that Mn and Co atoms in the S-LMO and L-LMO materials are in similar environments with quite similar local structures.

Figure 3(b,d) illustrate the Fourier-transformed EXAFS signals at the Mn and Co *K*-edges, respectively. There are two main peaks. The first peak corresponds to the Mn and Co adsorbing atoms that occupy octahedral sites, surrounded by six oxygen atoms (Mn-O for the Mn *K-*edge and Co-O for the Co *K-*edge). The second peaks are due to the interactions between Mn and Co adsorbing atoms and transition metal (TM) atoms in the transition metal layers (Mn-TM for Mn *K-*edge and Co-TM for Co *K-*edge). These peaks appear at similar positions (R_Mn−O_ = 1.60 Å, R_Mn−TM_ = 2.49 Å, R_Co−O_ = 1.49–1.51 Å, and R_Co−TM_ = 2.36 Å). However, the amplitudes of the Mn-TM and Co-TM peaks are noticeably different. The Mn-TM peak intensity of the S-LMO material is higher than that of the L-LMO material. In contrast, the Co-TM peak intensity of the L-LMO material is higher than that of the S-LMO material. This indicates that in this coordination shell, the Mn and Co have different types and numbers of neighboring species. It is notable that there is an equal amount of the Li_2_MnO_3_ and LiCoO_2_ components in both the materials. However, in the S-LMO material, the Li_2_MnO_3_ domain size is small, so it is more distributed. In the L-LMO material, the Li_2_MnO_3_ domain size is large, so the individual Li_2_MnO_3_ and LiCoO_2_ domains are more locally isolated. For the Mn-TM peak, its lower peak intensity is an indication of weaker X-ray scattering. This phenomenon results because the Mn atoms in the S-LMO material with a smaller Li_2_MnO_3_ domain size are surrounded by a larger number of Co atoms resulting in better scattering than Mn alone. There are 3 Li atoms and 3 Mn atoms in this coordination shell, and Li has a very low X-ray scattering power. This leads to weaker Mn-Mn/Li peak in the EXAFS spectra for the material with large Li_2_MnO_3_ domains. In the case of the Co *K*-edge, the Co-TM peak of the S-LMO material was lower than that of the L-LMO material. This is because the Co atoms in the 0.5Li_2_MnO_3_∙0.5LiCoO_2_ material with smaller Li_2_MnO_3_ domain size interacted with a larger number of Mn atoms, producing less scattering than Co. The XAS experimental results revealed that the 0.5Li_2_MnO_3_∙0.5LiCoO_2_ materials have the similar overall local structures with subtle differences in EXAFS spectra due to the different Li_2_MnO_3_ and LiCoO_2_ domain sizes, indicating different phase separation behaviors.

### Electrochemical characterizations

**Figure 4:**
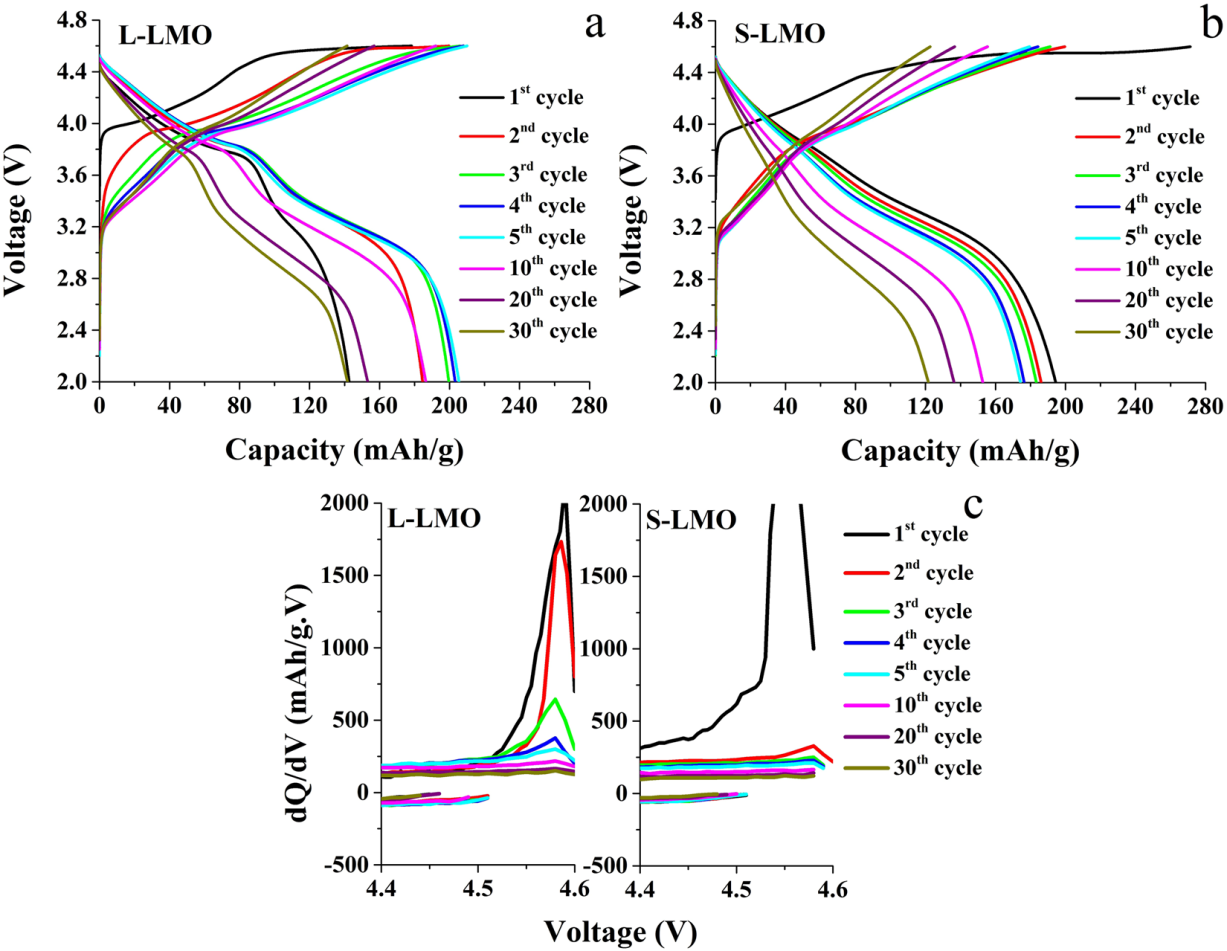
Voltage profiles (**a**,**b**) and differential capacity (**c**) of the 0.5Li_2_MnO_3_∙0.5LiCoO_2_ materials with larger Li_2_MnO_3_ domain (L-LMO) and smaller Li_2_MnO_3_ domain sizes (S-LMO).

A galvanostatic cycling technique was performed to examine the effect of various Li_2_MnO_3_ domain sizes on electrochemical properties of the 0.5Li_2_MnO_3_∙0.5LiCoO_2_ materials. The results are given in Fig. 4. The first charging voltage profiles (black curves) of both materials revealed two distinct plateaus at around 3.9 V and 4.5 V, as shown in Fig. 4(a,b). The first voltage plateau at 3.9 V corresponds to the oxidation of Co^3+^ to Co^4+^ in the LiCoO_2_ component. The second voltage plateau at 4.5 V corresponds to extraction of lithium ions from the structure of the Li_2_MnO_3_ component. Various charge theories have been introduced to describe the charge compensation mechanisms at this higher voltage plateau, including irreversible oxygen loss from the lattice. This contributes to large irreversible capacity loss^4,8,9^, and reversible oxygen redox, which induces reversible capacity^10–12^. However, Chen and Islam^50^ postulated that lithium ion extraction from the Li_2_MnO_3_ structure involves oxidation of oxygen, but the resulting oxygen holes (O^−^) are not thermodynamically stable. This leads to formation of an oxygen dimer, resulting in oxygen release from the lattice. The charge compensation mechanisms of the cathode materials in this study, occurring at 4.5 V, were dominated by an irreversible oxygen release from the Li_2_MnO_3_ structure revealing a high initial irreversible capacity loss. The charge compensation involving the oxygen release from the lattice always produces Li_2_O and layered MnO_2_ phases. The formation of the Li_2_O phase induces an increase in the initial charge capacity and a large irreversible capacity in the first cycle^4,36^. Moreover, the sloping discharge curves from 3.8 to 2.0 V resulted from intercalation of lithium ions into the layered MnO_2_ component^51^. The electrochemical reactions of these composite-based cathode materials can be described as follows. While lithium ions are de-intercalated from the LiCoO_2_ component at voltages of 3.9 V to 4.5 V, depleted lithium layers are formed in the LiCoO_2_ structure. Lithium ions in octahedral sites on lithium and manganese layers of the Li_2_MnO_3_ component diffuse into the depleted lithium to compensate for lithium ions in the LiCoO_2_ structure^4,52,53^. This compensation allows the overall structure of the cathode materials to remain stable during cycling. Nevertheless, during the discharge processes, lithium ions intercalate into the MnO_2_ component to form a layered LiMnO_2_ phase until the Li_2_MnO_3_ component is completely consumed. It is well established that the layered LiMnO_2_ phase often transforms into a spinel-like phase upon cycling, causing a large capacity and voltage drop as the cycle numbers increase. Controlling the activation of the Li_2_MnO_3_ component is an essential key to improving the cycling stability of composite-based cathode materials. Thus, strategies to retard this phase transformation from a layered Li_2_MnO_3_ phase to spinel-liked phases were introduced in several previous studies. These strategies include using suitable testing conditions (appropriate cut off voltage and current density)^3^ and controlling the Li_2_MnO_3_ domain size^54^. Ghanty *et al*. revealed that a large Li_2_MnO_3_ domain size can reduce the spinel phase transformation because a Li_2_MnO_3_ component with a large domain size is quite difficult to activate and produce a layered LiMnO_2_ phase. This leads to less spinel phase formation upon continuous cycling^54^.

Differential capacity plots obtained from differentiation of capacity as a function of its voltage profile for both materials at various cycle numbers at selected voltages ranging from 4.4 to 4.6 V are presented in Fig. 4(c). This was done to show more detail about the influence of the Li_2_MnO_3_ domain size on activation of the Li_2_MnO_3_ component during cycling. The oxidation peak between 4.5 V and 4.6 V corresponded to lithium and oxygen extraction from the Li_2_MnO_3_ component^55^. For the S-LMO material, the oxidation peak could not be clearly observed after the second cycle due to complete activation of the Li_2_MnO_3_ component after the second cycle resulting from its small domain size. In contrast, for the L-LMO material, an oxidation peak was clearly observed in subsequent cycles. In this material, the Li_2_MnO_3_ component was not completely activated in the first few cycles because the large Li_2_MnO_3_ domain size made lithium and oxygen extraction from the Li_2_MnO_3_ component was quite difficult. The presence of the oxidation peak suggested that Li_2_MnO_3_ activation still took place and the peak confirms the presence of a Li_2_MnO_3_ component during subsequent cycles. The remaining Li_2_MnO_3_ component after extended cycles stabilized the overall cathode structure during repeated cycling, resulting in higher cycling stability.

Lithium ion diffusion coefficients reflect the degree of lithium ion transport inside electrode materials. This is a crucial kinetic parameter for ion insertion/extraction in cathode materials. An understanding of lithium ion diffusion behaviors in electrode materials is very important to improve the electrochemical performance of lithium rich layered oxide cathode materials. A galvanostatic intermittent titration technique (GITT) was performed to study lithium ion diffusion behaviors in the 0.5Li_2_MnO_3_∙0.5LiCoO_2_ materials. It is well established that the lithium ion diffusion coefficient $({D}_{{{Li}}^{+}})$ of lithium ions can be determined by Fick’s 2^nd^ Law of Diffusion, which can be written as^56,57^:

1 $${D}_{{Li}^{+}}=\frac{{4}}{\pi }{(\frac{{m}_{B}{V}_{M}}{{M}_{B}S})}^{{2}}\,{(\frac{{\Delta E}_{S}}{\tau ({dE}_{\tau }/d\sqrt{\tau })})}^{{2}}\,(\tau \ll {L}^{{2}}/{D}_{{\mathrm{Li}}^{+}})$$

where *m*_*B*_ and *M*_*B*_ denote the molecular weight and mass of the active material, respectively. *V*_*M*_ is the molar volume of the prepared material, obtained from crystallographic data. *S* is the individual particle surface area of the electrode, and *L* is the thickness of the electrode. Given that the cell voltage profile (E) as a function of τ^1/2^ is quite linear (as shown in Fig. S3), Equation 1 can be further simplified^24,58^:

2 $${D}_{{{Li}}^{+}}=\frac{{4}}{\pi \tau }{(\frac{{m}_{B}{V}_{M}}{{M}_{B}S})}^{{2}}{(\frac{{\Delta E}_{S}}{{\Delta E}_{\tau }})}^{{2}}\,(\tau \ll {L}^{{2}}/{D}_{{{Li}}^{+}})$$

Indeed, the electrochemical reactions of lithium rich layered oxide cathode materials during cycling are very complex. They include lithium ion diffusion, oxygen loss, and a transition from a layered structure to a spinel form. Previously, Zhu and Wang^59^ used GITT to study the lithium ion diffusion coefficient in a LiFePO_4_ cathode material that exhibited a phase transformation upon cycling. Lithium ion diffusion coefficients of each phase (α and β phases) were separately determined to achieve a clear understanding of lithium ion diffusion behaviors in this material. However, the current study aims to investigate the overall lithium ion diffusion coefficients of 0.5Li_2_MnO_3_·0.5LiCoO_2_ cathode materials, which can be considered pseudo- or apparent diffusion coefficients^25,60^. Full details of this analysis for the lithium rich layered oxide cathode materials will be presented elsewhere (SK in preparation).

**Figure 5:**
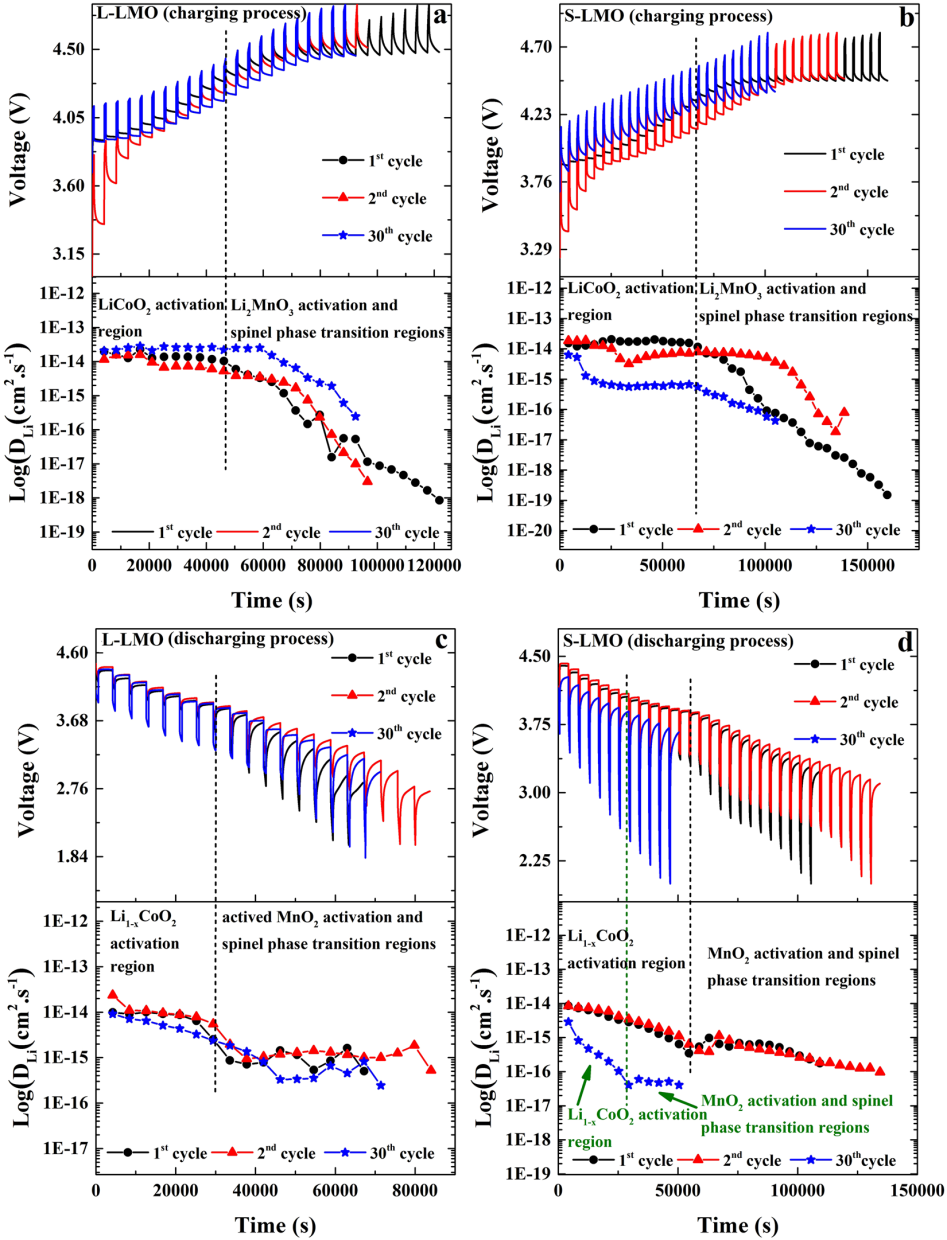
GITT profiles and calculated lithium ion diffusion coefficients during the 1^st^, 2^nd^ and 20^th^ cycles of the 0.5Li_2_MnO_3_∙0.5LiCoO_2_ materials with larger Li_2_MnO_3_ domain (L-LMO, **a**,**b**) and smaller Li_2_MnO_3_ domain sizes (S-LMO, **c**,**d**).

The voltage relaxation profiles using GITT and the calculated lithium ion diffusion coefficients during the charging and discharging processes of 0.5Li_2_MnO_3_·0.5LiCoO_2_ materials are shown in Fig. 5. During charging, two distinct diffusion coefficients can be observed. The first is believed to occur due to lithium ion extraction from the LiCoO_2_ structure and the second corresponds to lithium ion extraction from the Li_2_MnO_3_ structure accompanied by a structural transformation. During the initial charging process (voltages below 4.4 V), where lithium ions are extracted from the LiCoO_2_ component, the lithium ion diffusions coefficients are independent of the cell voltage showing constant values of around 10^−14^ cm^2^.s^−1^. At higher voltages (above 4.4 V), the lithium ion diffusion coefficients decreased rapidly reaching around 10^−19^ cm^2^.s^−1^ when the electrodes were charged to 4.8 V, corresponding to the lithium and oxygen extraction from the Li_2_MnO_3_ component. As can be seen, the lithium ion diffusion coefficients of the Li_2_MnO_3_ component are lower than those from the LiCoO_2_ component due to the low ionic conductivity of the Li_2_MnO_3_ component. Additionally, the structural transition from a layered Li_2_MnO_3_ component to a spinel form can induce large structural disorders and defects, which negatively impact lithium ion diffusivity. During discharge, the two distinct lithium ion diffusion coefficients were observed. They result from lithium ion insertion into the Li_1−x_CoO_2_ structure at higher voltages and activation of MnO_2_ (during the 1^st^ and subsequent cycles) and the transformed spinel phases (during the 2^nd^ and subsequent cycles) at lower voltages, respectively. The lithium ion diffusion coefficients resulting from the insertion of lithium ions into the Li_1−x_CoO_2_ structure showed higher values than those into the activated MnO_2_ and the spinel structures. This occurs due to the large structural disorders and defects formed during the spinel phase transition.

**Figure 6:**
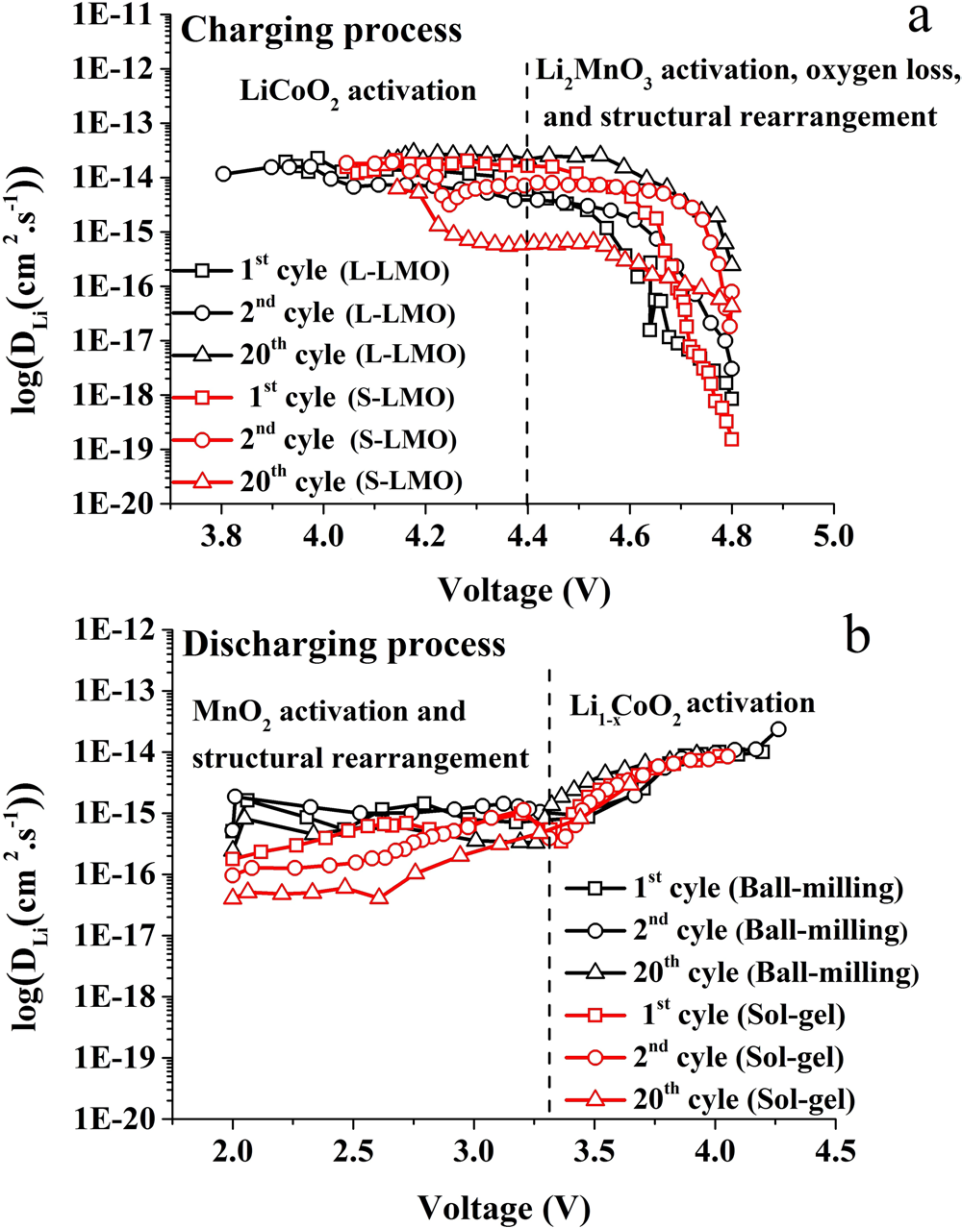
The effects of Li_2_MnO_3_ domain size on lithium ion diffusion coefficients as a function of cell voltage during charging (**a**) and discharging (**b**) of the 0.5Li_2_MnO_3_∙0.5LiCoO_2_ materials.

Figure 6 presents the calculated lithium ion diffusion coefficients as a function of cell voltage of the S-LMO and L-LMO materials. This figure compares the effect of the Li_2_MnO_3_ domain size on lithium ion diffusion coefficients of the 0.5Li_2_MnO_3_·0.5LiCoO_2_ materials. It is notable that the impact of the Li_2_MnO_3_ domain size on the electrochemical kinetics of the 0.5Li_2_MnO_3_∙0.5LiCoO_2_ materials can be clearly observed, especially in the voltage range of 4.4 to 4.8 V. The S-LMO material shows slightly higher lithium ion diffusion coefficients in this voltage range than the L-LMO material during the first two cycles. This result is not surprising because the smaller Li_2_MnO_3_ domain size provides shorter lithium ion diffusion paths, hence higher lithium ion diffusion coefficients. However, the difference is not dramatic because the average particle size of these two materials is quite similar, as can observed in Fig. 1. For extended cycles, the L-LMO material appears to have better lithium ion mobility, with lithium ion diffusion coefficients in this voltage range almost two orders of magnitude higher than the S-LMO material. These phenomena can be explained based on structural disorders and defects resulting during phase transformation of the Li_2_MnO_3_ component.

**Figure 7:**
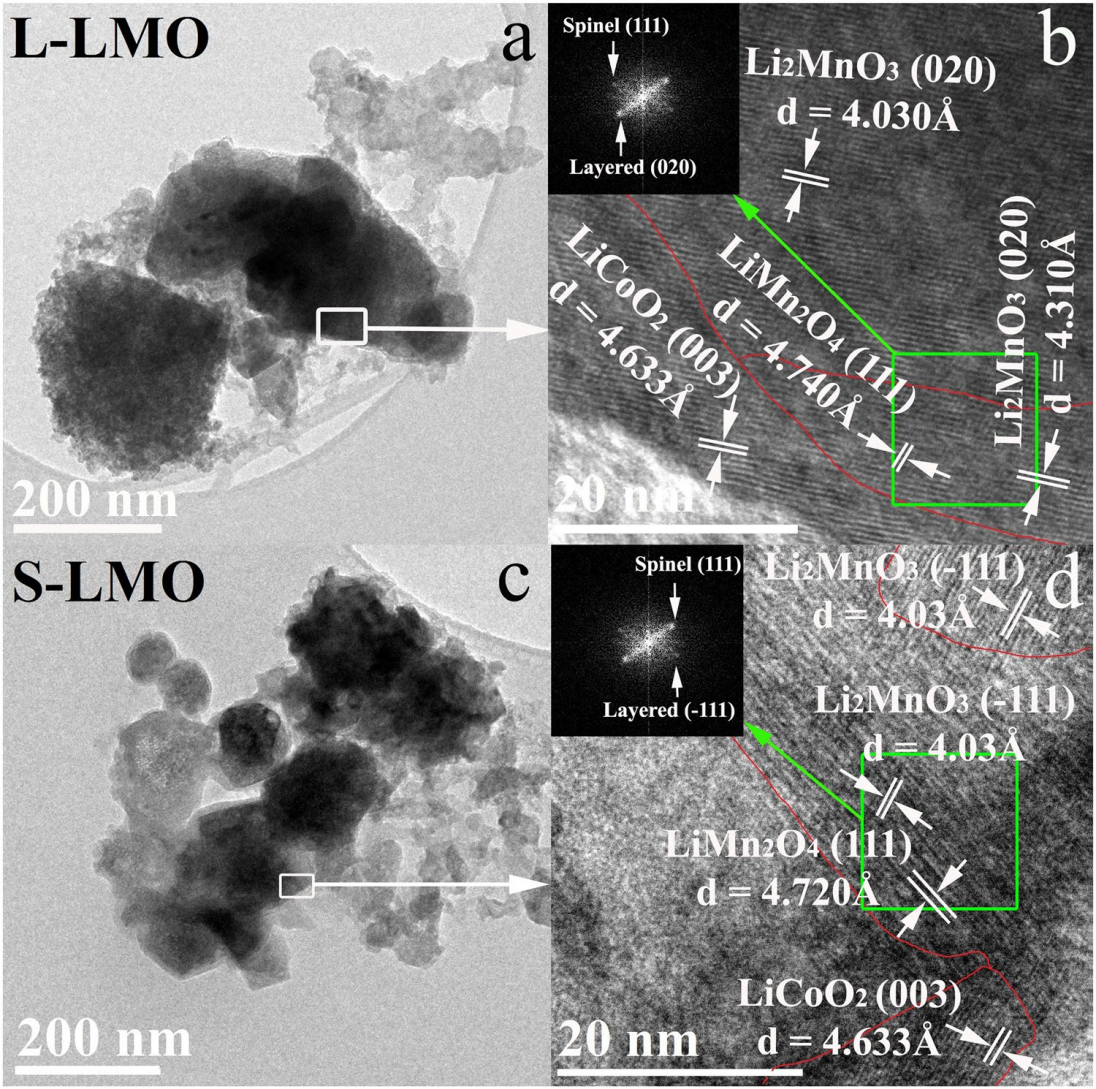
TEM and HRTEM images of the 0.5Li_2_MnO_3_∙0.5LiCoO_2_ materials with a larger Li_2_MnO_3_ domain size (L-LMO, **a**,**b**) and the 0.5Li_2_MnO_3_∙0.5LiCoO_2_ material with a smaller Li_2_MnO_3_ domain size (S-LMO, **c**,**d**) after cycling for 30 cycles.

Figure 7 shows high resolution transmission electron microscopy (HRTEM) images of the 0.5Li_2_MnO_3_∙0.5LiCoO_2_ materials after cycling for 30 cycles. Three main crystalline phases can be indexed in both materials. They are the Li_2_MnO_3_, LiCoO_2_, and spinel-like (LiMn_2_O_4_) domains. Additionally, the HRTEM image of the S-LMO material shows a large area with no lattice fringes, indicating significant formation of disorder phase(s) after extended cycling. The S-LMO material also appears to have greater spinel-like and disorder regions than the L-LMO material, as illustrated in Fig. 7(b,d). These defects act as barriers for lithium ion transport in the electrode materials. For the S-LMO material, the phase transition after Li_2_MnO_3_ phase activation induced a larger structural rearrangement, causing abundant lattice disorders and defects^24,27,28^. Moreover, in the absence the Li_2_MnO_3_ component after extended cycles, the overall structure of the cathode could not be stabilized, leading to degradation. These phenomena causes low lithium ion diffusion coefficients and poor cycling stability. The cycling stability and rate capability of the 0.5Li_2_MnO_3_∙0.5LiCoO_2_ materials with various Li_2_MnO_3_ domain sizes are presented in Fig. S4. The L-LMO material with larger Li_2_MnO_3_ domains can retard the formation of these structural imperfections during cycling better than the S-LMO material. This is reflected in higher lithium ion diffusion coefficients after 20 cycles. The GITT measurements and HRTEM images reveal the relationship between the Li_2_MnO_3_ component activation and lithium ion coefficients of a composite-based cathode material upon cycling. The results show that the 0.5Li_2_MnO_3_∙0.5LiCoO_2_ material with a large Li_2_MnO_3_ domain size can potentially retard spinel phase evolution during cycling with improved structural stability and electrochemical properties.

## Conclusions

Composite-based layered 0.5Li_2_MnO_3_∙0.5LiCoO_2_ cathode materials with various Li_2_MnO_3_ domain sizes were characterized and showed quite similar morphological, crystal and local atomic structures. However, the materials exhibited different phase separation behaviors. Lithium ion diffusion coefficients depend significantly on activation of the Li_2_MnO_3_ component, resulting in various cycling stabilities and levels of rate performance. The 0.5Li_2_MnO_3_∙0.5LiCoO_2_ material with larger Li_2_MnO_3_ domains shows a higher cycling stability and rate capability than the material with smaller Li_2_MnO_3_ domains. The Li_2_MnO_3_ domain size affects phase transition behaviors of the Li_2_MnO_3_ component after activation. For the first few cycles, the 0.5Li_2_MnO_3_∙0.5LiCoO_2_ material with a small Li_2_MnO_3_ domain size revealed higher lithium ion diffusion coefficients due to shorter lithium ion diffusion path lengths. However, after an extended number of cycles, the 0.5Li_2_MnO_3_∙0.5LiCoO_2_ material with the large Li_2_MnO_3_ domain size provided higher lithium ion diffusion coefficients, since the larger Li_2_MnO_3_ domain size can retard structural transitions. This leads to fewer structural rearrangements, reduced structural disorders and defects, which allow better lithium ion mobility in the material. The current work shows that controlling the Li_2_MnO_3_ component activation through an appropriate Li_2_MnO_3_ domain size is an effective strategy for improving both cycling stability and rate capability of lithium rich layered oxide cathode materials.

## Materials and Methods

### Cathode materials preparation

A sol-gel method was used to prepare Li_2_MnO_3_, LiCoO_2_, and 0.5Li_2_MnO_3_·0.5LiCoO_2_ materials. Raw materials included CH_3_COOLi·2H_2_O (Aldrich), Mn(CH_3_COO)_2·_4H_2_O (Aldrich), Co(CH_3_COO)_2_·4H_2_O (Aldrich) and ascorbic acid (with the molar ratio of metal ions to ascorbic acid of 2:1). To prepare each material, required amounts of the precursors were dissolved in ethyl alcohol and mixed with an aqueous solution of ascorbic acid under continuous stirring at a constant temperature of 80 °C until a viscous gel was formed. After that, the obtained gel was first pre-heated at 300 °C for 3 h to eliminate organic constituents, re-heated for crystallization at 800 °C in air for 10 h, and followed by furnace cooling to room temperature. Fine crystalline powders of Li_2_MnO_3_, LiCoO_2_, and 0.5Li_2_MnO_3_·0.5LiCoO_2_ (S-LMO) materials were obtained.

Another 0.5Li_2_MnO_3_·0.5LiCoO_2_ material was synthesized by a ball**-**milling route. Stoichiometric amounts of the Li_2_MnO_3_ and LiCoO_2_ materials prepared in the previous step. The mixed powders and zirconia balls (weight ratio of ball to powder was 20:1) were placed in 90 mL of ethanol in a sealed Teflon bottle. Then, the mixed powders were ball milled using a rotational speed of 180 rpm for 72 h. The obtained mixture was evaporated overnight at 80 °C in a vacuum drying box, followed by final firing at 800 °C in air for 10 h, and followed by furnace cooling to room temperature. Fine crystalline powder of 0.5Li_2_MnO_3_·0.5LiCoO_2_ material with larger Li_2_MnO_3_ domain sizes (L-LMO) was obtained.

### Structure and morphology characterization

X-ray diffraction (XRD) (Empyrean, PANalytical) was employed to study the crystal structure of the materials using Cu-*K*α radiation operated at 45 kV and 40 mA. The data were recorded with a step size of 0.003° and a step time of 30 s/step over a 2θ range from 15° to 70° using Ni as a filter. X-ray absorption spectroscopy (XAS) (BL. 2.2, SLRI, Thailand) was performed to investigate the local atomic structure of the prepared materials. Morphology of the 0.5Li_2_MnO_3_·0.5LiCoO_2_ materials and the presence of Li_2_MnO_3_ and LiCoO_2_ domains were observed using transmission electron microscopy (TEM) (JEOL, JEM-2100 Plus).

### Electrochemical measurements

To prepare the electrodes, 78 wt% of synthesized materials, 11 wt% of Super P carbon black (Alfa Aesar) as a conductive additive, and 11 wt% of polyvinylidene fluoride as a binder (PVDF, Arkema) dissolved in N-methyl-2-pyrollidone (NMP, Aldrich) solvent were mixed using a horizontal shaker for 2 h to form a slurry. The obtained slurry was coated on an Al foil by a doctor blade technique and dried overnight in vacuum at 80 °C. Swagelok type cells were fabricated in an Ar- filled glovebox using Li metal foil as an anode (Alfa Aesar), 1 M LiPF_6_ dissolved in ethyl carbonate (EC), dimethyl carbonates (DMC), and diethyl carbonate (DEC) (4:3:3 in volume) (MTI) as an electrolyte, and Celgard 2400 as a separator. Cycling stability of the prepared electrodes was studied using a galvanostatic charge/discharge test (BST8-MA, MTI) with a voltage window of 2.0–4.6 V and current rate of C/5 at 30 °C. A galvanostatic intermittent titration technique (GITT) (WBCS-300, WonATech) was performed to study lithium ion diffusion behaviors in the 0.5Li_2_MnO_3_·0.5LiCoO_2_ cathode materials, using a voltage window of 2.0–4.8 V.

## Electronic supplementary material

Supplementary information
