# Supplementary material for: Structural and Electrochemical Kinetic Properties of 0.5Li2MnO3∙0.5LiCoO2 Cathode Materials with Different Li2MnO3 Domain Sizes
Source: Sci Rep. 2019 Jan 23;9:427. doi: 10.1038/s41598-018-36593-9 (PMC6344595; doi:10.1038/s41598-018-36593-9)
Supplement: Supplementary file 1 — Supplementary information [file 41598_2018_36593_MOESM1_ESM.pdf]

Supplementary information

Structural and Electrochemical Kinetic Properties of $0.5Li_2MnO_3 \cdot 0.5LiCoO_2$ Cathode Materials with Different Li_2MnO_3 Domain Sizes

Songyoot Kaewmala,[a] Wanwisa Limphirat,[b] Visittapong Yordsri,[c] Hyunwoo Kim,[d] Shoaib Muhammad,[d] Won-Sub Yoon,[d] Sutham Srilomsak,[a,e] Pimpa Limthongkul,[c,*] and Nonglak Meethong [a,e,*]

[a]Materials Science and Nanotechnology Program, Department of Physics, Faculty of Science, Khon Kaen University, Khon Kaen, 40002, Thailand

[b]Synchrotron Light Research Institute, Nakhon Ratchasima, 30000, Thailand

[c]National Metal and Materials Technology Center, National Science and Technology Development Agency, Pathumthani, 12120, Thailand

[d]Department of Energy Science, Sungkyunkwan University, Suwon, 16419, Republic of Korea

[e]Institute of Nanomaterials Research and Innovation for Energy (IN-RIE), Research Network of NANOTEC- KKU (RNN), Khon Kaen University, Khon Kaen, 40002, Thailand

*Corresponding authors: nonmee@kku.ac.th (N.M.) and pimpal@mtec.or.th (P.L.)

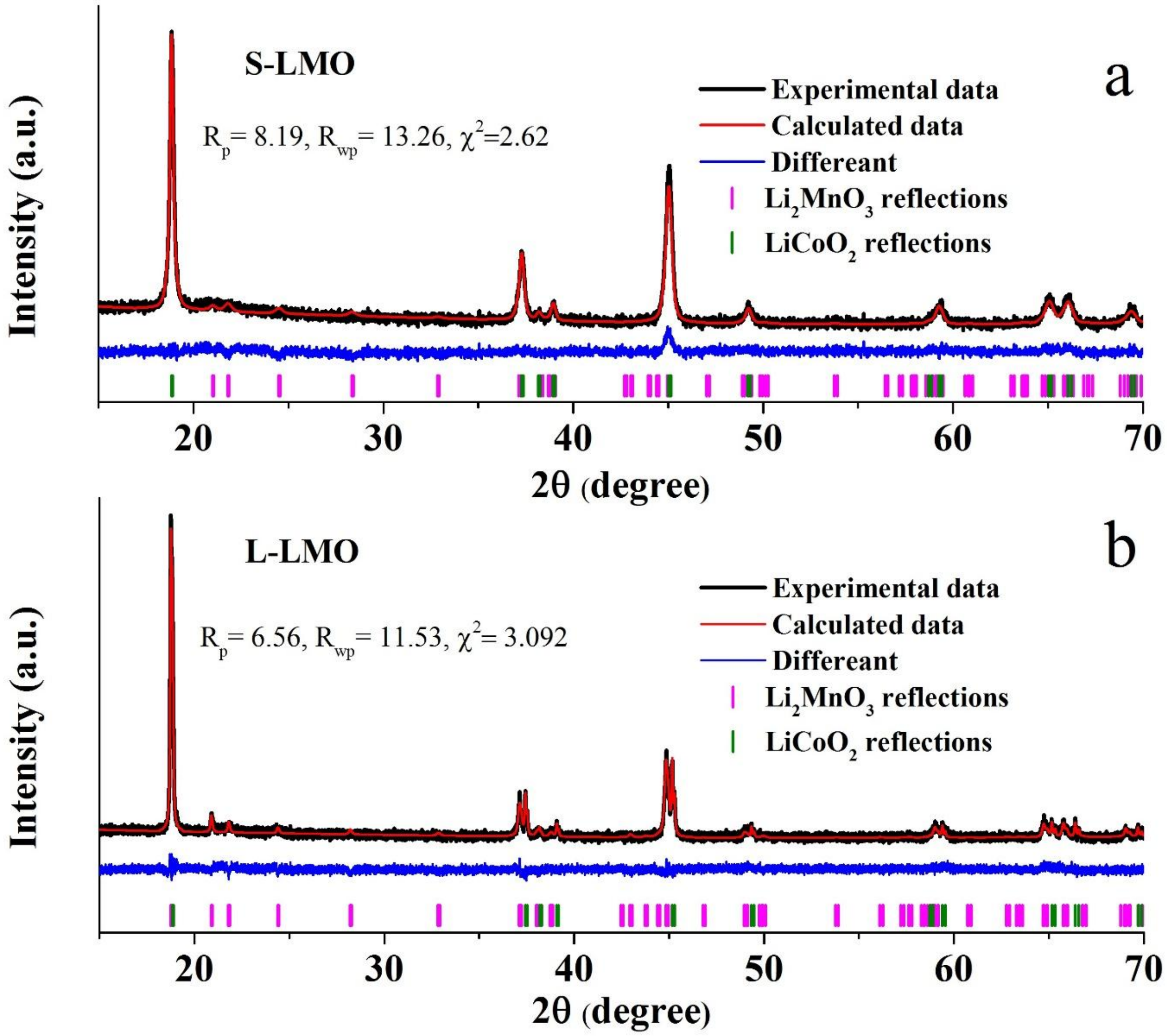

Fig. S1 Rietveld refinements of the $0.5Li_2MnO_3·0.5LiCoO_2$ materials synthesized using ball-milling (a) and sol-gel (b) approaches

Table S1 Lattice parameters obtained using Rietveld refinements

Samples	$LiCoO_2$ ($R\bar{3}m$) α=β=90°, γ=120°			Li_2MnO_3 (C2/m) α=γ=90°				
	a and b (Å)	c (Å)	Unit cell volume (Å^3)	a (Å)	b (Å)	c (Å)	β (°)	Unit cell volume (Å^3)
L-LMO	2.815 ±0.000	14.143 ±0.001	97.072	4.919 ±0.000	8.513 ±0.001	5.011 ±0.000	109.068 ±0.010	198.318
S-LMO	2.830 ±0.000	14.161 ±0.002	98.188	4.919 ±0.002	8.504 ±0.004	4.992 ±0.002	108.961 ±0.051	197.453

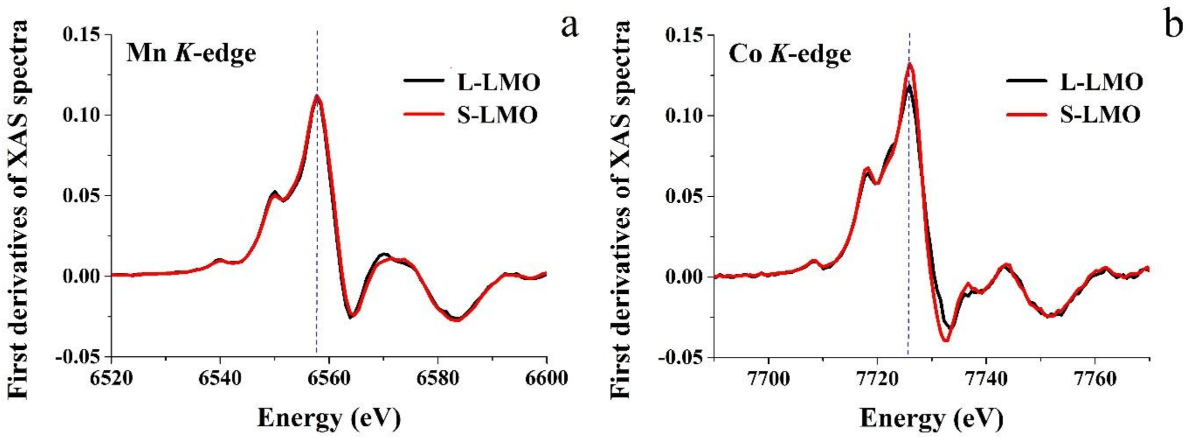

Fig. S2 The first derivatives of the XAS spectra at Mn (a) and Co (b) *k*-edges of $0.5Li_2MnO_3{\cdot}0.5LiCoO_2$ materials using ball-mill and sol-gel approaches.

Fig. S3 (a) presents a selected titration curve at 4.08 V during the first charging process of an electrode made from the L-LMO material. As presented in Fig. 5(b), the overall relationship between E and $\tau^{1/2}$ is quite linear. However, it exhibits a non-linear behavior at voltages less than 4.11 V, where structural transformation from a layered to a spinel structure during the charging process occurs. Indeed, the electrochemical reactions of the lithium rich layered oxide cathode materials during cycling are very complex. They include lithium ion diffusion, oxygen loss, and a structural transition. Full details of each of these contributions to GITT profiles for the lithium rich layered oxide cathode materials will be presented elsewhere (SK in preparation).

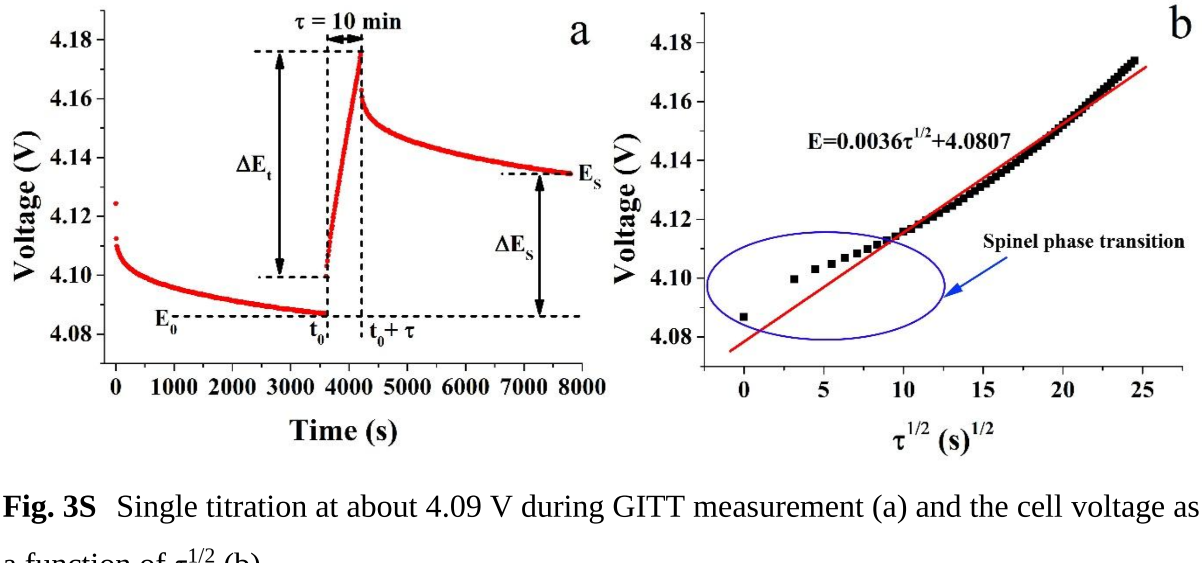

Fig. 3S Single titration at about 4.09 V during GITT measurement (a) and the cell voltage as a function of $\tau^{1/2}$ (b)

Cycling stabilities and columbic efficiencies of the $0.5Li_2MnO_3{\cdot}0.5LiCoO_2$ materials with larger Li_2MnO_3 domain (L-LMO) and smaller Li2MnO3 domain (S-LMO) sizes are illustrated in Fig. 4S(a). Both cathodes offered low columbic efficiencies during the first few cycles because lithium and oxygen were extracted from the Li_2MnO_3 component, leading to a large initial irreversible capacity. The initial discharge capacities of the cathode with the smaller Li_2MnO_3 domain size were higher than the cathode with the larger Li_2MnO_3 domain size. This promoted Li_2MnO_3 activation, leading to a higher discharge capacity in the first few cycles. However, the discharge capacity of the L-LMO material was low and then increased slowly for the first few cycles. This resulted from the large Li_2MnO_3 domain size, which retarded Li_2MnO_3 activation. The Li_2MnO_3 activation in the L-LMO material still occurred over extended cycles until the entire Li_2MnO_3 component was completely consumed, bringing about an increased discharge capacity in the first few cycles. The L-LMO material also exhibited higher cycling stability because of its larger Li_2MnO_3 domain. This effectively reduced spinel phase evolution upon repeated cycling. Moreover, the remainder of the Li_2MnO_3 component in subsequent cycles induced the overall structure of the cathode material was stabilized by the Li_2MnO_3 component during continuous cycling. The rate capabilities of the S-LMO and L-LMO materials are presented in Fig. 4S(b). The S-LMO material exhibited a lower rate performance than the L-LMO material because of its smaller Li_2MnO_3 domain size that induced greater Li_2MnO_3 phase activation. This led to a large structural rearrangement, bringing about abundant lattice disorder and defects, which greatly retarded lithium ion diffusion when cycle numbers were increased as shown in the GITT results. This led to lower rate performance in the S-LMO material.

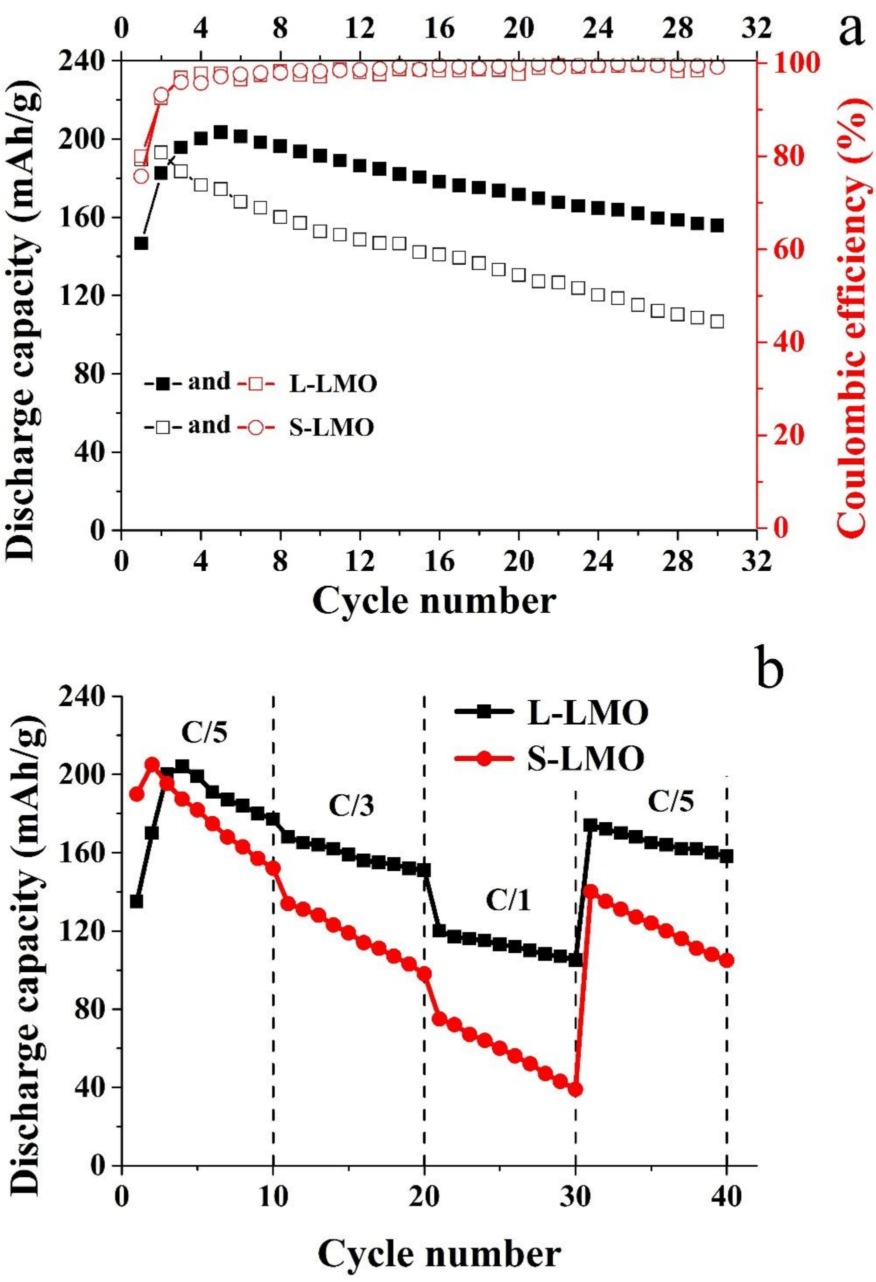

Fig. 4S Cycling stabilities and columbic efficiencies (a) and rate capability (b) of the $0.5Li_2MnO_3{\cdot}0.5LiCoO_2$ materials with various Li_2MnO_3 domain sizes.
